# Assessment of quality of life in patients with knee osteoarthritis

**DOI:** 10.1590/1413-785220152306150596

**Published:** 2015

**Authors:** Marcio Massao Kawano, Ivan Luis Andrade Araújo, Martha Cavalcante Castro, Marcos Almeida Matos

**Affiliations:** 1 Escola Bahiana de Medicina e Saúde Pública, Salvador, Bahia, Brazil.

**Keywords:** Osteoarthritis, Knee, Quality of life

## Abstract

**OBJECTIVE:**

: To assess the quality of life of knee osteoarthritis patients using the SF-36 questionnaire

**METHODS:**

: Cross-sec-tional study with 93 knee osteoarthritis patients. The sample was categorized according to Ahlbӓck score. All individuals were interviewed with the SF-36 questionnaire

**RESULTS:**

: The main finding of the study is related to the association of edu-cation level with the functional capacity, functional limitation and pain. Patients with higher education level had better functional capacity when they were compared to patients with basic level of education

**CONCLUSION:**

: Individuals with osteoarthritis have a low perception of their quality of life in functional capacity, functional limitation and pain. There is a strong association between low level of education and low perception of quality of life. **Level of Evidence IV, Clinical Case Series.**

## INTRODUCTION

Chronic diseases of the musculoskeletal system are among the most prevalent health hazards in the world's population. 1 Of these, knee osteoarthritis is a major public health issue related to age, characterized by progressive loss of articular cartilage resulting in pain, functional impairment, disability and diminished patient's quality of life. 2 About 10% of the population over 60 years old complain of this condition. 3 In the US, about 37% of the population aged above 60 present a diagnosis of knee osteoarthritis. ^4^ It is estimated that in 2025 the prevalence of knee osteoarthritis will increase by 40% due to the aging of the world population. ^1^ This data becomes even more alarming in Brazil, since the current Brazilian population over 60 years old is 19 million and it is estimated that it will increase to 64 million by 2050.^4^

Some risk factors contribute to the appearance of the disease, such as gender, age, trauma, overuse and genetic conditions. The main tissues affected by osteoarthritis is the synovium, bone and hyaline cartilage.^5^ It is a joint disease that begins with cartilage degeneration and gradually affects periarticular soft tissues and the subchondral bone, producing chronic in-flammation with synovitis, osteophytosis, loss of joint space, bone remodeling and ultimately, it progresses to severe and irreversible joint destruction.^6^

Patients with knee osteoarthritis tend to increase their physi-cal limitations, pain and functionality restriction with disease progression. 7 Thus, these individuals suffer from progressive increasead impact on their activities of daily living, which leads to losses in labor relations, leisure, social life, and sleeping qua-lity, leading also to important decrease in their quality of life. 7 Thus, an important outcome to be evaluated in patients with knee osteoarthritis is the quality of life of these individuals. Nor-mally, quality of life is evaluated as the impact the disease cau-ses to the subject. According to the World Health Organization, "quality of life" is described as an individual's perception of his/ her position in life in the context of the culture and value syste-ms in which he lives and in relation to his goals, expectations, standards and concerns. 8

Some previous studies have evaluated quality of life in patients with knee osteoarthrosis. 3 8 11 However, the mostly used ques-tionnaires of quality of life and disease impact are the World Health Organization Quality of Life Group (WHOQOL-100), WHOQOL-Bref, Western Ontario and McMaster Universities Osteoarthritis Index (WOMAC), Arthritis Impact Measurements Scale (AIMS), and OA Knee and Hip QoL (OAKHQOL). Howe-ver, some of these questionnaires deal specifically with osteoar-thritis, failing to measure important aspects of patients' mental, social and emotional health.

This study aims to evaluate the quality of life of a group of patients with knee osteoarthrosis with the Medical Outcomes Study-36 - Item Short -Form Health Survey (SF-36). This questionnaire is short and easy to administer and understand; moreover, since it is a generic instrument, it enables comparisons of the impact of quality of life in knee osteoarthritis with other health conditions, also allowing correlation with psychosocial aspects.

## METHODS

This is a cross-sectional, descriptive and analytical study with a sample of 93 patients treated in the Outpatient of Knee Surgery Service at *Hospital Santa Izabel, Santa Casa de Misericórdia da Bahia*, Salvador, Brazil, from December 2012 to May 2013. In-dividuals were recruited by sequential non-probability sampling among those who met the study's inclusion criteria.

To calculate the sample size we considered an estimated of 37% in the prevalence of patients with functional dependence of osteoarthritis. ^4^ Alpha type error was set at 5% and the power of the test at 90%. Using the formula to estimate a population para-meter, the sample size was estimated at 90 individuals. Inclusion criteria were: individuals who had medical diagnosis of unilateral or bilateral knee osteoarthritis aged between 40 and 70 years old, both genders, without neurological disorders, who agreed to sign the Free and Informed Consent form. Individuals who had any central nervous system alteration, cognitive impairment, who had undergone previous knee surgeries or other diseases as-sociated with the osteoarticular system (rheumatic or, metabolic bone diseases, etc.), as well as degenerative diseases, which could affect the quality of life and functional independence of the subject, such as cancer, heart disease, Parkinson's disease, among others, were not included in the study.

Patients were initially seen by the attending physician and spe-cific conduct was adopted according to the unit's clinical cri-teria, not being influenced by the research protocol. Then, the patients were referred for inclusion or not in the study. Those patients who understood the objectives of the study and agre-ed to participate were inserted in the study after signing the Free and Informed Consent form. The research protocol was approved by the Institutional Research Ethics Committee, under protocol number 158465/2012.

The participants of the survey were evaluated by data collection from medical records and by specific instruments of the study. General and clinical data were collected through a question-naire with socio-demographic data such as gender, age, race, marital status, religion, profession, education level, occupation, medical diagnosis and disease duration. After collecting data from patients, a specialist orthopedist (qualified by the Brazilian Society of Knee Surgery) carefully evaluated each patient's knee osteoarthritis. The condition was radiographically stratified according to Ahlbӓck's rating system. 12

Stratification consists of a radiographic evaluation in antero-posterior (AP) and profile (P) views obtained with the patient in standing position and monopodal support. Classification is described as follows. Grade 1: moderate cartilage destruction (narrowing of the joint space) in AP; Grade 2: total destruction of cartilage (obliteration or near obliteration of the joint space) in AP; Grade 3: wear of the tibial plateau smaller than 5mm in AP; Grade 4: wear of the tibial plateau 5-15mm in AP; and Grade 5: wear greater than 15mm of tibial plateau with severe subluxation of the tibia in AP.^12^ The Ahlbӓck classification of osteoarthritis was later defined into categories: mild/moderate and severe. Mild/moderate degree was regarded as Ahlbӓck's grade 1, 2 and 3 (generally with conservative treatment indication), and serious as Ahlbӓck's grades 4 and 5 (indication of surgical treatment).

The evaluation of the quality of life of the patients was measu-red using the Medical Outcomes Study 36 - Item Short-Form Health Survey (SF-36). 13 This instrument consists of 36 items, grouped into eight domains: functional capacity, physical as-pects, pain, general health, vitality, social aspects, emotional aspects and mental health. For each subject and for each of the eight dimensions we obtained a score upon applying a measurement scale with values ​from zero (which corresponds to the worst health status) to 100 (best health status).^13^ SF-36 was applied in the form of a structured interview, the questions were read by the interviewer seeking maximum exemption in obtaining the answers.

### Statistical analysis

Initially the variables were checked for normal distribution. La-ter, the data were presented in frequency distribution tables in the case of categorical variables and mean and standard deviation in the case of numerical variables. For the analysis, age, gender, marital status, religion, race, level of education, main occupation, profession and medical diagnosis were con-sidered as independent variables. The variable regarded as dependent was quality of life measured by SF-36 question-naire. The association between dependent and independent variables was performed using the chi-square test in case of categorical variables. Student's *t*-test and ANOVA were used for comparison of numeric variables. To verify differences in the analysis of variance, *post-hoc* Bonferroni's test was used. To identify independent predictors in the SF-36, a model with multivariate analysis of MANOVA was used, adopting the Ba-ckwards technique. Within the model, variables with *P* <0.05 were adopted and named independent predictors. The model calibration was performed by the determination coefficient R2. For all analyzes the statistical significance adopted was 5%. The statistical program SPSS version 20 was used for all analyzes.

## RESULTS

In total, 93 individuals (69 females) participated in the study. The mean age of the sample was 61.2 years old and the time of diag-nosis of knee osteoarthritis was 8.1 years. Table 1 presents data on sample characteristics. Table 2 shows the mean and standard deviation of the eight domains of the SF-36 applied to the sample. Table 3 contains the comparison of the characteristics of the sample and domains of SF-36 questionnaire. There was a sta-tistically significant difference for the characteristic level of edu-cation (patients with more education had better score) in the areas of functional capacity, functional limitations and pain. In addition, the occupation characteristics (being active or retired) and degree of osteoarthritis (mild/moderate and severe) sho-wed also a statistically significant difference between groups for the functional capacity domain.

There was a statistically significant difference between groups regarding education up to elementary school (29.1 ± 20.1) and higher education (61.2 ± 30.0) in the functional capacity domain (*P* = 0.001), between elementary school (14.3 ± 25.2) and higher education (45.3 ± 44.9) in the functional limitation domain (*P* = 0.009); and between elementary school (26.2 ± 17.7) and higher education (44.9 ± 27.2) in the pain domain

**Table 1:**
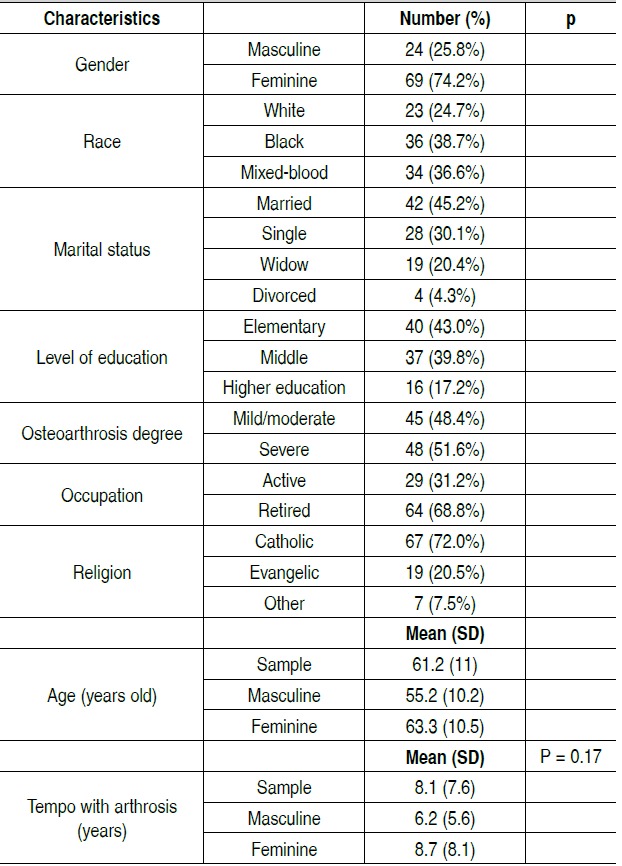
. Characteristics of participants with knee osteoarthritis (n=93).

SD: Standard Deviation.

**Table 2:** Mean and Standard deviation of SF-36 domains in the sample.

Domains of SF-36	Mean (±SD)
Functional Capacity	37.1 (±27.1)
Functional Limitation	25.1 (±35.3)
Pain	32.9 (±23.1)
General Health Status	54.6 (±19.1)
Vitality	48.7 (±24.1)
Social Aspects	50.1(±29.3)
Emotional Aspects	38.6 (±42.3)
Mental Health	60.1 (±27.3)

SD: Standard Deviation.

(*P* = 0.01). Regarding occupation there was no difference be-tween active (45.8 ± 29.3) and retirees (33.2 ± 25.4) in the functional capacity domain (*P* = 0.03). Moreover, regarding osteoarthritis level there were no differences between mild/ moderate (42.2 ± 30.2) and severe (32.3 ± 23.2) also in the functional capacity domain (*P* = 0.05).

In the multivariate analysis, initially three dependent variables were verified: functional capacity, pain, and functional limitation. As covariates, Ahlbӓck's degree of osteoarthritis, age, time of osteoarthritis diagnosis, gender, level of education, religion, oc-cupation, marital status and race were selected. There were no independent predictor factors on the dependent variables func-tional limitation and pain, however, the only predictor in functional capacity was level of education with *P* <0.001; R2 = 0.11.

## DISCUSSION

The aim of this study was to verify through the SF-36 ques-tionnaire the perception of quality of life in patients with knee osteoarthritis. Our results showed that patients with knee osteo-arthritis have a low perception of their quality of life, especially in the fields functional capacity, functional limitations and pain. We also found that there is a strong association between low educational level and low quality of life in this group of indivi-duals. The participants mostly reported being retired (68%) and there was a statistically significant difference between active and retired participants. In the functional capacity domain of SF-36 active participants obtained an average score of 45.8, and pensioners, 33.2 (*P* = 0.03). We also observed that 51.6% of participants had a diagnosis of severe osteoarthritis, revealing that over half of the sample had surgical indication according to Ahlbӓck's rating.

The profile of the sample showed a higher number of female individuals. A fraction of 74.2% of patients with knee osteoarthritis were women. This data coincides with published findings that show that osteoarthritis of the knee has a higher incidence and prevalence in females. 3 14 In this study, despite the vast majo-rity of female participants, there were no statistically significant differences according to gender in the values obtained​ for the quality of life domains assessed by SF-36. The mean age of 61.2 ± 11 years old in this sample taken together with the average time of diagnosis of the disease (8.1 ± 7.6 years) is worrisome. This information suggests that the studied population had been diagnosed before 60 years old. The early manifestation of symp-toms interferes directly on productivity, labor and treatment costs. 15 On average, spending on knee osteoarthritis per patient can reach up to € 871 per month. Of this amount, 83% is associated

with loss of productivity costs and 17% to direct treatment costs. Thus, it can be inferred that human, social and health costs can be even higher in populations diagnosed before the age of 60, as it was the case in our sample.

**Table 3:**
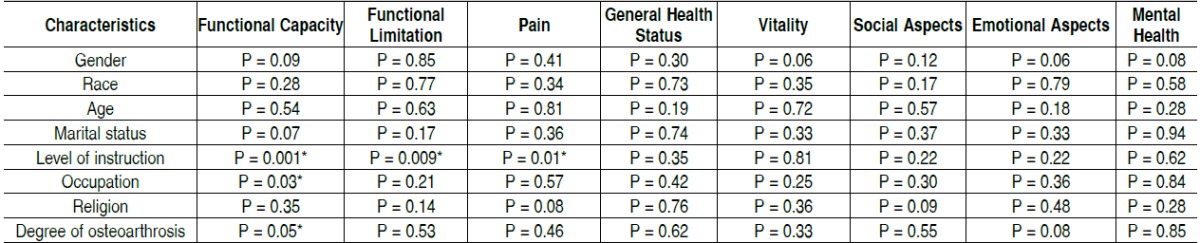
Comparison of results of SF-36 domains according to sample's characteristics (P values).

*Statistically significant difference

An important finding of this study was the evidence of a statis-tically significant difference between the level of education and the domains of quality of life on SF-36 questionnaire: functional capacity, pain and functional limitation. Patients with higher education had better functional capacity when compared to elementary school-educated patients. From our data, we con-cluded that functional limitation was also dependent on the level of education. Moreover, the SF-36 pain domain was also statistically significant when comparing low educational level to higher education. The scores in these three areas of SF-36 demonstrate better quality of life in those with higher education. This information helps us to infer that low educational level may account for the negative impact on quality of life of these patients, since there is little information about the prevention and treatment of osteoarthritis, rather exhaustive and greater impact work activities that could expose the individual to risk factors for development of osteoarthritis. Table 4 confirms these findings, since it is also possible to observe an association between level of education and degree of osteoarthritis. The results show that the lower the educational level, the greater the impairment of osteoarthritis and the greater the impact of this condition on quality of life of the subjects.

Multivariate analysis also confirms this finding by showing that the level of education worked as the only independent predictor of functional capacity in the patients studied. Previous studies indicate a relationship between low education and the prevalence of knee osteoarthritis. In the study of Alkan et al., 16 about 70% of the study participants had low-middle education, resulting in poor quality of life in this group. In another study, 17 the authors found that low educational level increases up to twice the chance of having osteoarthritis, and therefore a low perceived quality of life. According to these authors, usually individuals with low education have manual occupational acti-vities or repetitive physical labor. In the same study, as well as low education, other risk factors for developing osteoarthritis were age over 60, obesity, physical labor and feminine gender. Within the functional capacity domain, it was found that indivi-duals with a more advanced degree of osteoarthritis (often with surgical indication) had worse scores. These data corrobora-te several previous studies 9 11 17 which also showed that the greater the degree of osteoarthrosis, the lower the perceived quality of life for individuals with this joint disease. As this is an open cross-sectional study, it was not possible to determine the impact of all variables as compared to the general popu-lation. Other variables that could also be considered as risk or confounding factors could not be included in the study, such as body mass index, profession, level of physical activity, fa-mily history, and comorbidities. However, this was not the main goal of this study, and it would not be possible to cover all the complex variables influencing osteoarthritis. Since this is an exploratory study in outpatients, it has good internal validity and moderate external validity, but its relevance is justified because it represents one of the few Brazilian studies on the subject.

**Table 4:** Association between level of education and level of os-teoarthrosis.

Level of Education	Level of Osteoarthrosis		P	Mild/Moderate	Severe
Elementary	15	25
Middle	17	20	0.01
Higher education	13	3

Chi-square test.

## CONCLUSION

Individuals with osteoarthritis have a low perception of their quality of life in the domains functional capacity, functional li-mitations and pain. There is a strong association between low educational level and low quality of life. This finding was also related to the fact that individuals with low education are enga-ged in physical work activities and higher impacts.
